# Epidemiological shift and clinical characteristics of rhinovirus genotypes in acute respiratory tract infection cases in Kunming, China, from 2019 to 2023

**DOI:** 10.3389/fcimb.2025.1678343

**Published:** 2025-11-14

**Authors:** Guiqian Zhang, Limei Ba, Luping Liu, Ting Su, Hongyan Zhu, Ya Xu, Yufan Zhang, Yafei Huang, Xin Fan, Jinxiu Wan, Jianmei Gao, Hailong Li, Min Su

**Affiliations:** 1 Department of Clinical Laboratory, The First People’s Hospital of Yunnan Province, The Affiliated Hospital of Kunming University of Science and Technology, Kunming, China; 2 Institute of Basic and Clinical Medicine, Yunnan Provincial Key Laboratory for Clinical Virology, The First People’s Hospital of Yunnan Province, Kunming, China; 3 Department of Clinical Laboratory, Yunnan Honghe Prefecture Central Hospital, Gejiu, China

**Keywords:** rhinovirus, genotype, epidemiological shift, clinical characteristics, Kunming

## Abstract

**Background:**

We aimed to investigate the impact of the coronavirus disease 2019 (COVID-19) outbreak on the genetic diversity and clinical severity of rhinovirus (RV) in acute respiratory tract infection (ARTI) cases in Kunming, China.

**Methods:**

A total of 25,769 patients with ARTI from September 1, 2019 to December 31, 2023 were enrolled. Patients with COVID-19 were excluded from the study. Nasopharyngeal swabs were collected and screened for RV using multiplex reverse transcription polymerase chain reaction. RV-positive samples underwent nested PCR targeting the VP4/VP2 region. The amplified products were sequenced for genotype identification.

**Results:**

A total of 1,017 RV-positive cases were identified (detection rate 3.95%; 1,017/25,769), and all three RV species—RV-A, RV-B, and RV-C—were detected; RV-A was the most prevalent. Overall, 128 distinct RV genotypes, 40 untyped strains, and 1 recombinant strain (A1B) were identified. The predominant genotypes differed between age groups: those <18 years were mainly infected with C-untyped, A36, and A12, whereas those ≥18 years had C1, B52, and A60. Among RV-positive cases, 42.97% were associated with lower respiratory tract infections (LRTI), mainly caused by RV-A and RV-C. RV-C was associated with higher C-reactive protein and procalcitonin. Co-occurrences were recorded in 24.98% (254/1,017) of RV-positive cases and were associated with prolonged hospitalization. RV exhibited no clear seasonal pattern; however, following the COVID-19 outbreak, RV-C was replaced by RV-A.

**Conclusions:**

RV remains endemic with significant genetic diversity in the region. The COVID-19 pandemic led to a notable shift in the dominant circulating species from RV-A to RV-C. Emerging mutant strains have contributed to increased disease severity. Both RV-C and RV-A contribute to LRTI in children.

## 1 Introduction

Acute respiratory tract infection (ARTI) is the most common infectious disease globally and the leading cause of mortality among children under 5 years, ranking first among incidences of acute infectious diseases worldwide (Walker et al., 2013; Liu et al., 2015; Muehling et al., 2018). In developed countries, approximately 80% of ARTIs are viral, with rhinovirus (RV) identified as a primary pathogen. Beyond the common cold, RV is closely associated with pediatric pneumonia, bronchitis, wheezing, asthma exacerbations, and worsening of pre-existing respiratory conditions in adults (Mahony, 2008; Vandini et al., 2019; Biagi et al., 2020). In China, viral infection rates of up to 46.9% have been reported in children aged ≤ 5 years; RV has the highest detection rate in pediatric cases of pneumonia and pneumonia in school-age children (Li et al., 2021). In children, RV causes 20–40% of bronchiolitis cases and 50–80% of wheezing and asthma exacerbations (Jartti et al., 2019; Bergroth et al., 2020).

During COVID-19, many restrictive public health measures were implemented worldwide, which dramatically impacted the spread of other common respiratory pathogens. The Chinese government established a prevention and control policy involving restricting the flow of people in January 2020 (Wang et al., 2023; Chen et al., 2023). Precise prevention and control have prevented the spread of the new coronavirus. On December 7, 2022, the COVID-19 prevention and control measures were fully lifted. With the increase in opportunistic infections, the types and prevalence of common respiratory pathogens have changed (Zhang et al., 2024). Elucidating the complex interactions between viruses, bacteria, and virus-bacteria combinations can help understand the epidemiological characteristics of respiratory pathogens and plan public health strategies for infection control.

RV is a non-enveloped virus belonging to the genus *Enterovirus* within the family *Picornaviridae* (Jacobs et al., 2013). Molecular studies classify RV into three species—RV-A, RV-B, and RV-C—based on amino acid homology in the P1, 2C, and 3CD regions, as well as nucleotide homology in the VP4/VP2 region. To date, 169 subtypes have been identified, including RV-A80, RV-B32, and RV-C57 genotypes (Bochkov and Gern, 2012; Palmenberg and Gern, 2015). These genotypes differ in virulence and infection patterns, resulting in varied clinical severities. RV-A is more commonly associated with upper respiratory tract infections (URTIs) in adults. Conversely, RV-C predominates in pediatric respiratory infections, while RV-B is frequently detected in asymptomatic individuals (Esneau et al., 2022), underscoring the importance of accurate RV genotyping for clinical severity assessment.

RV circulates year-round, affecting all age groups, with children being particularly susceptible due to their immature immune systems (Busse et al., 2010; Lopes et al., 2020). Consequently, RV infections are significantly more prevalent in children under 10 (Takashita et al., 2021; Jia et al., 2022). Kunming, the capital of Yunnan Province in southwestern China, has a population of approximately 8.46 million and is a multiethnic border city. This is the first large-scale investigation on RV in Kunming spanning the COVID-19 pandemic (2019-2023), with unique comparative analysis across pre-, during-, and post-pandemic periods, aimed at investigating the epidemiological patterns and clinical features of RV during this unique timeframe. The findings can provide more evidence of its role as a significant respiratory pathogen.

## 2 Methods

### 2.1 Study population

This study was conducted at a large tertiary general hospital in Kunming, China, which has over 2,000 inpatient beds and an annual patient volume exceeding 2.5 million. From September 1, 2019, to December 31, 2023, 25,769 patients meeting the diagnostic criteria for ARTI were consecutively enrolled. Patients with COVID-19 were excluded from the study. Among them, 22,277 were hospitalized cases and 3,492 were outpatients. The study population comprised 14,410 males and 11,359 females, with ages ranging from 1 day to 123 years. The inclusion criteria were (1) no clinical treatment administered prior to sample collection and (2) a diagnosis of ARTI. ARTIs were defined as illnesses presenting with (1) onset within 7 days; (2) at least two respiratory symptoms, including nasal discharge, nasal obstruction, sneezing, sore throat, cough, malaise, chills, and headache; and (3) a Jackson score of ≥ 2 (Jackson et al., 1962). Exclusion criteria included a history of chronic pulmonary diseases, cystic fibrosis, interstitial lung disease, congenital heart disease, immunodeficiency, or malignancy. RV-positive samples were classified into three groups—before (2019, 09–2020, 01), during (2020, 01–2022, 12), and after COVID-19 (2023, 01–2023, 12)—based on the national timeline of the outbreak and subsequent public health policy changes in China (Chen et al., 2023). Further stratification was performed by age into nine subgroups: <1 year, 1–3 years, 4–6 years, 7–17 years, 18–24 years, 25–34 years, 35–44 years, 45–59 years, and ≥60 years.

The classification of URTI and LRTI was performed according to the International Statistical Classification of Diseases and Related Health Problems (ICD-10-WHO) and the diagnoses and information documented in the patients’ records. Patients with rhinitis, pharyngitis, and laryngitis were classified as having a URTI, whereas patients with bronchitis, bronchiolitis, and pneumonia (characterized by cough, wheezing, and/or dyspnea) were classified as having an LRTI (Neugebauer et al., 2022).

The Medical Ethics Committee of Yunnan First People’s Hospital (Approval Number: KHLL2023-KY055) approved the study, and it was conducted in strict accordance with the Declaration of Helsinki. Informed consent was obtained from the parents or guardians of all participants. Data were stored and analyzed anonymously.

### 2.2 Sample collection and nucleic acid extraction

For each participant, one nasopharyngeal swab sample was collected either on the day of the clinic visit or the following day and stored at 4 °C in a sterile sample tube containing cell preservation solution (HEALTH Gene Technologies Co., Ltd., Ningbo, China). All testing procedures were completed within 24h of sample collection. Nucleic acid was extracted from clinical samples using a Nucleic Acid Extraction or Purification Kit (HEALTH Gene Technologies Co., Ltd., Ningbo, China), and the extracted nucleic acid was stored at –80 °C. An automated nucleic acid extraction system (Smart LabAssist-32; Taiwan Advanced Nanotech Inc., Taiwan) was employed.

### 2.3 Pathogen detection

All participants completed the multiplex PCR test, and detection was performed using a Multiple Detection Kit for Thirteen Respiratory Pathogens (13× kit, HEALTH Gene Technologies Co., Ltd.). The instruments used for pathogen detection included a polymerase chain reaction (PCR) thermal cycler (A300, Hangzhou LongGene Scientific Instruments Co., Ltd., China) and an Applied Biosystems 3500 Dx analyzer (Thermo Fisher Scientific, USA). The detected pathogens included Influenza virus types A (InfA), InfA-H1N1pdm09, InfA-H3N2, influenza virus types B, human respiratory syncytial virus, human parainfluenza virus, human coronavirus, human rhinovirus, human metapneumovirus (HMPV), bocavirus (Boca), human adenovirus, chlamydia (including *Chlamydia trachomatis* and *Chlamydia pneumoniae*), and *Mycoplasma pneumoniae* (MP).

Bacterial and fungal pathogens were cultivated using standard microbiological techniques and were considered co-infections if detected in the samples.

### 2.4 Genotype identification

Specific primers targeting the VP4/VP2 region of the RV genome were designed for RV-positive samples according to a previously published protocol (Wisdom et al., 2009). Primers were synthesized by Sangon Biotech (Shanghai) Co., Ltd. Nested PCR was performed to amplify the target gene. For the first-round PCR, the primers used were F: CCGGCCCCTGAATGYGGCTAA and R: ACATRTTYTSNCCAAANAYDCCCAT. Amplification was performed using the PrimeScript™ One Step RT-PCR Kit Ver. 2 (TaKaRa). The reaction mixture contained 1 μL Enzyme Mix, 12.5 μL 2× 1 Step Buffer, 1 μL forward primer, 1 μL reverse primer, 2 μL template RNA, and 7.5 μL RNase-free dH_2_O. Thermal cycling conditions were as follows: 50 °C for 30min (one cycle); 94 °C for 2min (one cycle); 94 °C for 30 s, 56 °C for 30 ssec, and 72 °C for 1min (35 cycles); followed by a final extension at 72 °C for 5min (one cycle). For the second-round PCR, the primers used were: ACCRACTACTTTGGGTGTCCGTG and R: TCWGGHARYTTCCAMCACCANCC. Amplification was conducted using GoTaq^®^ Master Mixes (Promega). The reaction mixture included 15 μL 2× GoTaq Green Master Mix, 1 μL forward primer, 1 μL reverse primer, 1 μL template from the first-round PCR, and 12 μL RNase-free dH_2_O. Thermal cycling conditions were 94 °C for 3min (one cycle); 94 °C for 30 s, 55 °C for 30 s, and 72 °C for 45 s (35 cycles); followed by a final extension at 72 °C for 5min (one cycle). PCR products were validated by capillary electrophoresis. Samples yielding a specific band near 500 bp were sent to Sangon Biotech (Shanghai) Co., Ltd. for sequencing. Sequences were aligned and genotypes identified using NCBI BLAST (http://blast.ncbI.nlm.nih.gov/Blast.cgi). The final fragment used for analysis was 458 bp in length.

### 2.5 Statistical analysis

Comparative analyses were conducted based on the study period and age group. Statistical analyses were performed using SPSS statistical software (version 26.0; SPSS, Inc., Chicago, USA), and plotting was performed using OriginPro 2021. We determine the data distribution through normality tests. Measurement data that follow a normal distribution are expressed as mean ± standard deviation; measurement data that do not follow a normal distribution are expressed as median and interquartile range (IQR), rate, or percentage composition. For comparisons between groups that do not follow a normal distribution, nonparametric Mann–Whitney U text is used. Count data were expressed as the number of cases and percentages, and the χ2 test was used for group comparisons. A p-value of < 0.05 was considered statistically significant, with a significance level of α = 0.05 (bilateral).

## 3 Results

**Figure 1:**
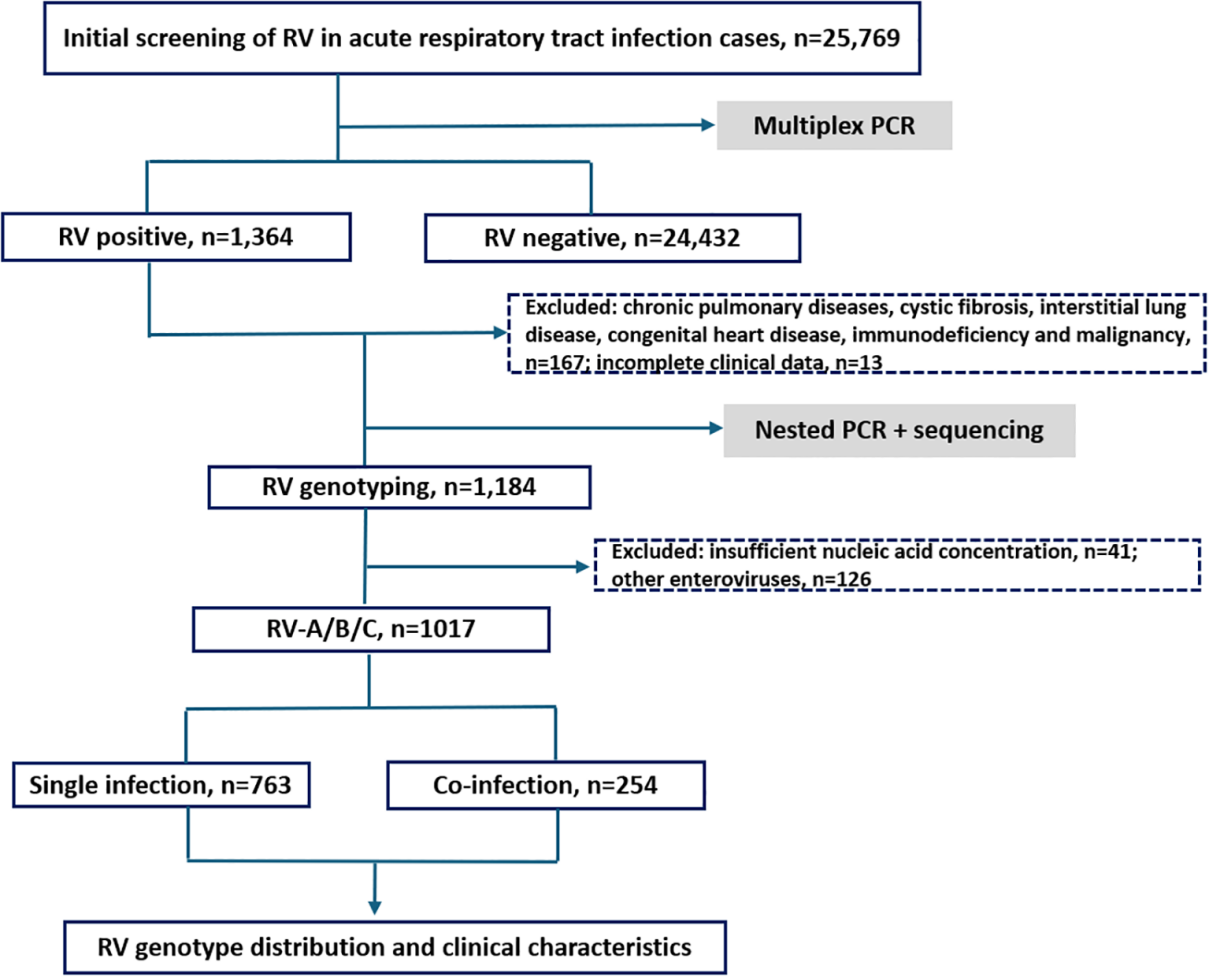
Research flowchart.

**Figure 2:**
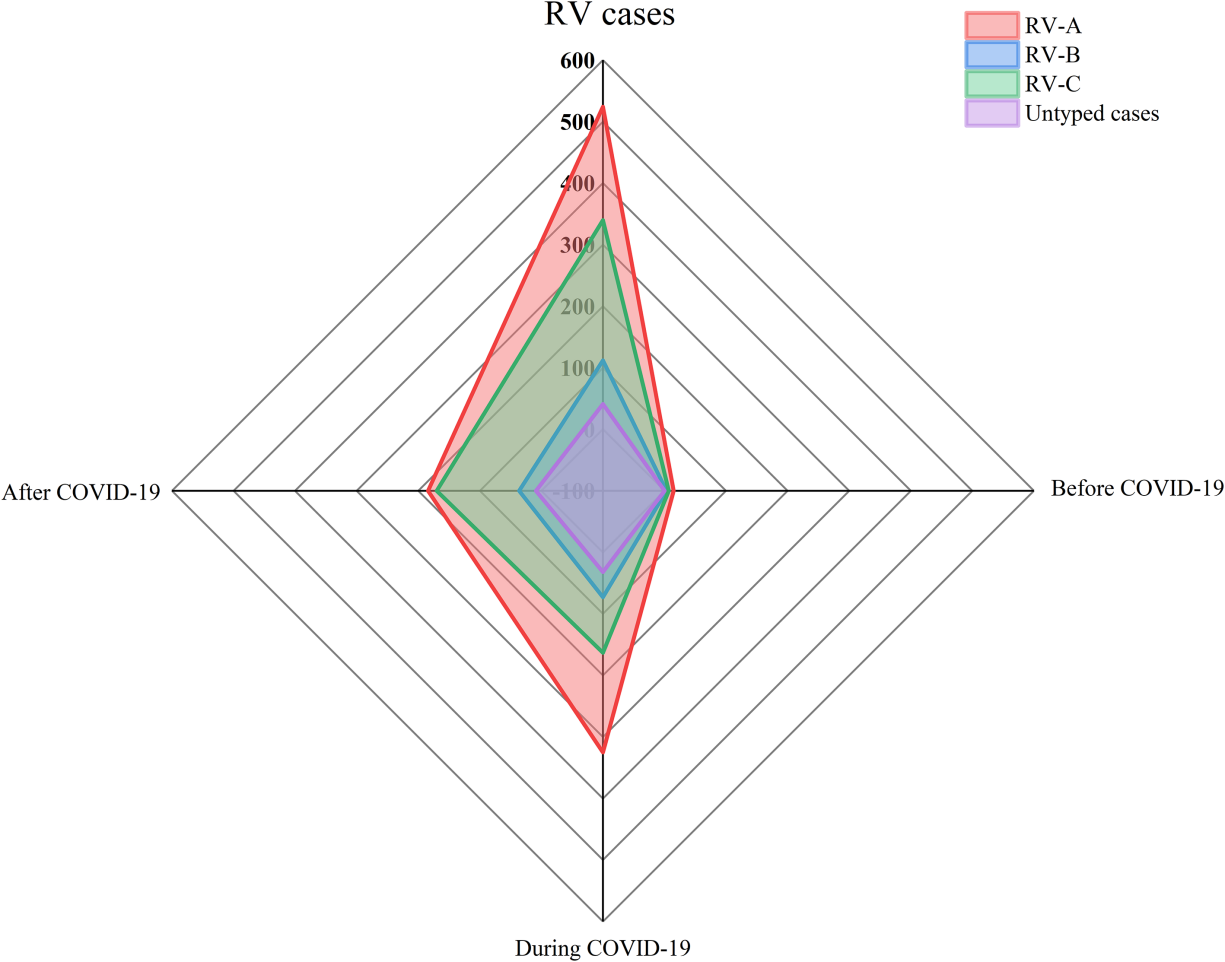
Comparison of rhinovirus genotype detections before COVID-19, during COVID-19 and after COVID-19. Pathogens detected by type, with *n* indicating the frequency of detection. The period before COVID-19 is from September 2019 to January 2020, the period during COVID-19 is from January 2020 to December 2022, and the period after COVID-19 is from January 2023 to December 2023.

Among the 25,769 samples screened, a total of 1,017 RV samples were confirmed and successfully genotyped, yielding a detection rate of 3.95% (1,017/25,769). The RV screening and selection process is illustrated in the corresponding flowchart. All three RV species — RV-A, RV-B, and RV-C were detected, with RV-A being the most prevalent (52.31%, 532/1,017), followed by RV-C (36.68%, 373/1,017), and RV-B (11.01%, 112/1,017). The distribution differences among the three groups were statistically significant (P < 0.05). The study population ranged in age from 1 day to 123 years. The median age of individuals infected with RV-B (12 years) was significantly higher than that of RV-A and RV-C cases (both 5 years). The detection rate among hospitalized patients was 4.29% (955/22,277), notably higher than that observed in outpatients at 1.78% (62/3,492). Additionally, the detection rate was slightly higher in males (4.02%, 579/14,410) than in females (3.86%, 438/11,359) (see **Figures 1**, **2** and **Table 1**).

**Table 1:** Study population and clinical features of RV cases.

Characteristics		RV-A	RV-B	RV-C	Total	*P*
Study population						
Positive	[% (n/total)]	2.06 (532/25769)	0.43 (112/25769)	1.45 (373/25769)	3.95 (1017/25769)	**0.000**
proportion	[% (n/total)]	52.31%(532/1017)	11.01%(112/1017)	36.68%(373/1017)	100 (1017/1017)	**0.000**
female	[% (n/total)]	1.99 (226/11359)	0.47 (53/11359)	1.40 (159/11359)	3.86 (438/11359)	**0.000**
male	[% (n/total)]	2.12 (306/14410)	0.41 (59/14410)	1.48 (214/14410)	4.02 (579/14410)	**0.000**
age(years)	median (IQR)	5(2-30)	12(3-38)	5(2-23)	6(2-30)	**0.005**
outpatients	[% (n/total)]	0.92 (32/3492)	0.69 (24/3492)	0.17 (6/3492)	1.78 (62/3492)	**0.000**
inpatients	[% (n/total)]	2.25 (501/22277)	0.39 (88/22277)	1.62 (366/22277)	4.29 (955/22277)	**0.000**
length of stay(days)	median (IQR)	7(6-11)	9(6-13)	9(6-11)	8(6-11)	0.123
ICU stay	[median range]	0.49 (5/1017)	0.29 (3/1017)	0.49 (5/1017)	1.28 (13/1017)	0.734
Clinical presentation						
fever	[% (n/total)]	22.03 (224/1017)	4.62 (47/1017)	15.73 (160/1017)	42.38 (431/1017)	**0.000**
Cough	[% (n/total)]	30.19 (307/1017)	5.80 (59/1017)	20.85 (212/1017)	56.83 (578/1017)	**0.000**
runny nose	[% (n/total)]	5.70 (58/1017)	1.38 (14/1017)	4.82 (49/1017)	11.90 (121/1017)	**0.000**
rhinobyon	[% (n/total)]	2.46 (25/1017)	0.29 (3/1017)	2.26 (23/1017)	5.01 (51/1017)	**0.000**
pharyngalgia	[% (n/total)]	2.16 (22/1017)	0.59 (6/1017)	2.16 (22/1017)	4.92 (50/1017)	**0.005**
wheeze	[% (n/total)]	4.42 (45/1017)	0.88 (9/1017)	2.65 (27/1017)	7.96 (81/1017)	**0.000**
dyspnea	[% (n/total)]	2.75 (28/1017)	0.79 (8/1017)	1.47 (15/1017)	5.01 (51/1017)	**0.002**
arthralgia	[% (n/total)]	0.79 (8/1017)	0.39 (4/1017)	0.98 (10/1017)	2.16 (22/1017)	0.277
convulsion	[% (n/total)]	1.18 (12/1017)	0.29 (3/1017)	1.08 (11/1017)	2.56 (26/1017)	0.059
rash	[% (n/total)]	0.09(1/1017)	0.00(0/1017)	0.79(8/1017)	0.88(9/1017)	**0.002**
Diagnosis						
URTI	[% (n/total)]	12.49 (127/1017)	3.15 (32/1017)	9.64 (98/1017)	25.27 (257/1017)	**0.000**
pharyngitis	[% (n/total)]	4.33 (44/1017)	1.47 (15/1017)	5.31 (54/1017)	11.11 (113/1017)	**0.000**
tonsillitis	[% (n/total)]	5.41 (55/1017)	1.28 (13/1017)	3.15 (32/1017)	9.83 (100/1017)	**0.000**
rhinitis	[% (n/total)]	2.06 (21/1017)	0.20 (2/1017)	0.59 (6/1017)	2.85 (29/1017)	**0.000**
laryngitis	[% (n/total)]	0.69 (7/1017)	0.20 (2/1017)	0.59 (6/1017)	1.47 (15/1017)	0.245
LRTI	[% (n/total)]	23.89 (243/1017)	3.54 (36/1017)	15.54 (158/1017)	42.97 (437/1017)	**0.000**
pneumonia	[% (n/total)]	16.03 (163/1017)	2.26 (23/1017)	10.03 (102/1017)	28.32 (288/1017)	**0.000**
bronchiolitis	[% (n/total)]	5.21 (53/1017)	1.08 (11/1017)	3.83 (39/1017)	10.13 (103/1017)	**0.000**
asthma	[% (n/total)]	1.57 (16/1017)	0.10 (1/1017)	1.08 (11/1017)	2.75 (28/1017)	**0.002**
COPD	[% (n/total)]	1.08 (11/1017)	0.10 (1/1017)	0.59 (6/1017)	1.77 (18/1017)	**0.015**
imageological change	[% (n/total)]	47.82 (209/437)	9.61 (42/437)	30.66 (134/437)	88.10 (385/437)	**0.000**
Undefined	[% (n/total)]	15.93 (162/1017)	4.33 (44/1017)	11.50 (117/1017)	31.76 (323/1017)	**0.000**
Laboratory testing						
White blood cell	median (IQR)	9.51(6.39-11.12)	8.92(5.67-11.26)	9.29(6.22-11.57)	9.36(6.26-11.22)	0.485
CRP	[mean ± SD]	7.6 ± 28.76	12.39 ± 30.53	15.05 ± 41.65	10.85 ± 34.39	**0.012**
PCT	[mean ± SD]	11.42 ± 28.81	3.99 ± 23.01	10.32 ± 30.77	10.27 ± 29.02	0.096
Co-infection	[% (n/total)]	12.19 (124/1017)	3.15 (32/1017)	9.64 (98/1017)	24.98 (254/1017)	**0.000**
viral	[% (n/total)]	6.00 (61/1017)	1.28 (13/1017)	5.70 (58/1017)	12.98 (132/1017)	**0.000**
bacterial	[% (n/total)]	4.03 (41/1017)	1.47 (15/1017)	1.87 (19/1017)	7.37 (75/1017)	**0.000**
fungal	[% (n/total)]	0.29 (3/1017)	0.00 (0/1017)	0.39 (4/1017)	0.69 (7/1017)	0.155
Others	[% (n/total)]	1.18 (12/1017)	0.39 (4/1017)	2.85 (29/1017)	4.42 (45/1017)	**0.000**

URTI, upper respiratory tract infection; LRTI, lower respiratory tract infection; COPD, chronic obstructive pulmonary disease; RV, rhinovirus; CRP, C-reactive protein; PCT, procalcitonin; ICU, Intensive Care Unit. Reference range of CRP:0-5mg/L; Reference range of PCT:0-0.05ng/L; IQR: inter-quartile range.

*Statistical significance: p < 0.05. The bold values represent significant results with P < 0.05.

**Figure 3:**
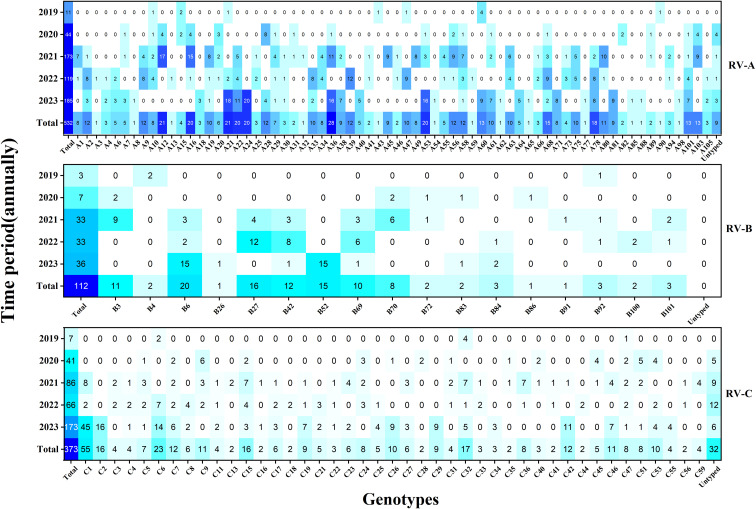
Heat map of the total number of rhinovirus genotypes detected each year during the study period (n = 1017). Darker colors indicate higher detection frequencies.

Here, a total of 128 distinct and identified RV genotypes were detected, along with 40 untyped strains and one recombinant strain (A1B). Among these, RV-A included eight untyped strains and the single A1B recombinant strain, while RV-C included 32 untyped strains. Of the 128 classified genotypes, 68, 17, and 43 were RV-A, RV-B, and RV-C, respectively. The most frequently identified genotypes during the COVID-19 period followed the pattern A12 > A16 > B27 > C-untyped > A36. Conversely, the dominant genotypes in the after COVID-19 period demonstrated a shift to C1 > C2 > A24 > A21 > A53.(see **Figure 3**).

**Figure 4:**
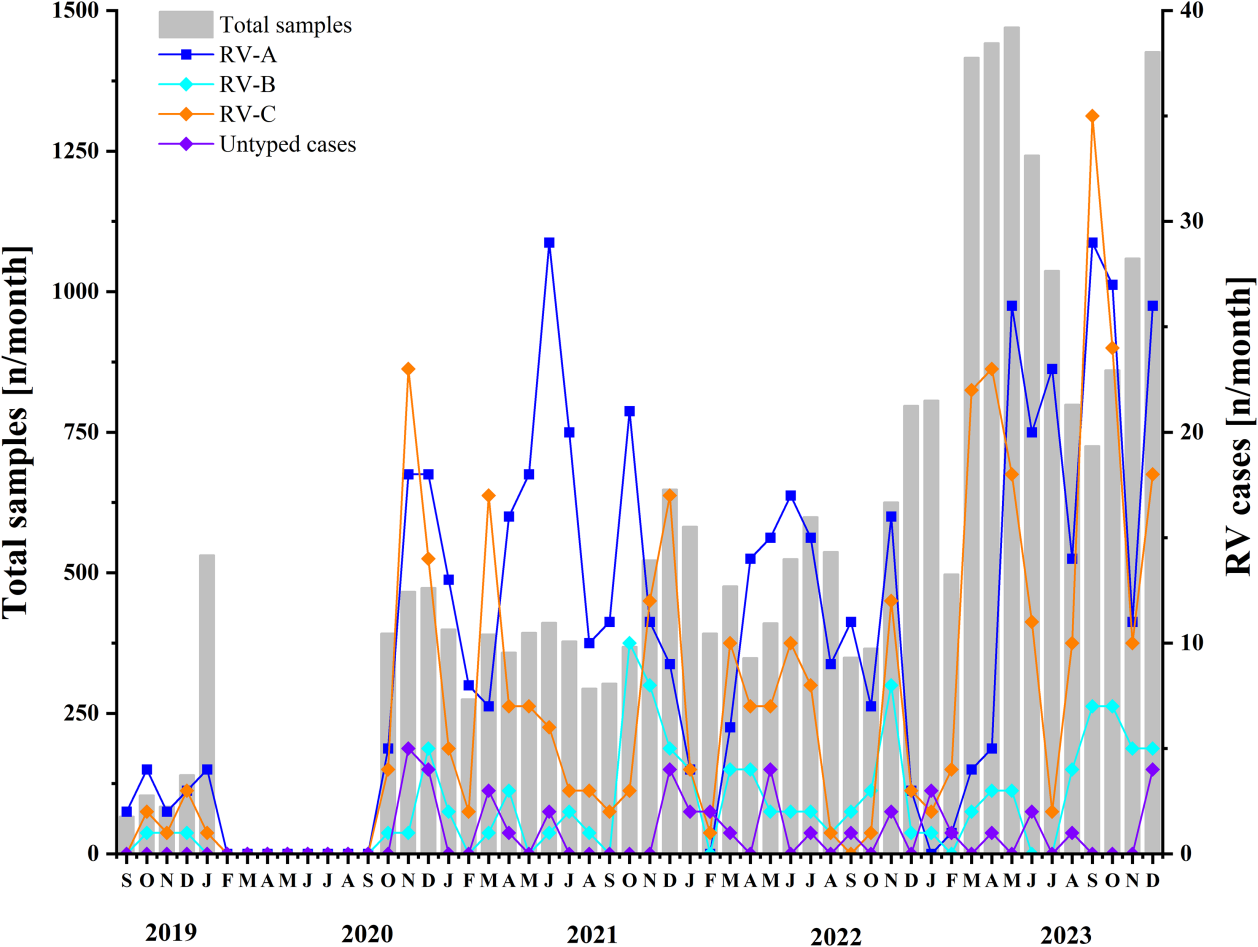
Monthly distribution of samples included between 2019 and 2023. Two y-axes are used: the left axis represents the total samples in this study, while the right axis indicates the number of detected rhinovirus A, rhinovirus B, and rhinovirus C cases, as well as untyped rhinoviruses. The period before COVID-19 is from September 2019 to January 2020, the period during COVID-19 is from January 2020 to December 2022, and the period after COVID-19 is from January 2023 to December 2023.

Following the COVID-19 pandemic, the predominant circulating genotype underwent a notable shift from RV-A to RV-C. Prior to COVID-19, RV activity reached its peak in October. Throughout the COVID-19 period, RV-A exhibited peak activity in July and November, whereas RV-C demonstrated the highest prevalence in March and November, with minimal activity recorded in February. In the post-COVID-19 period, RV circulation reached its maximum in September and declined to its lowest point in January. (see **Figure 4**, **Supplementary Table 1**).

**Figure 5:**
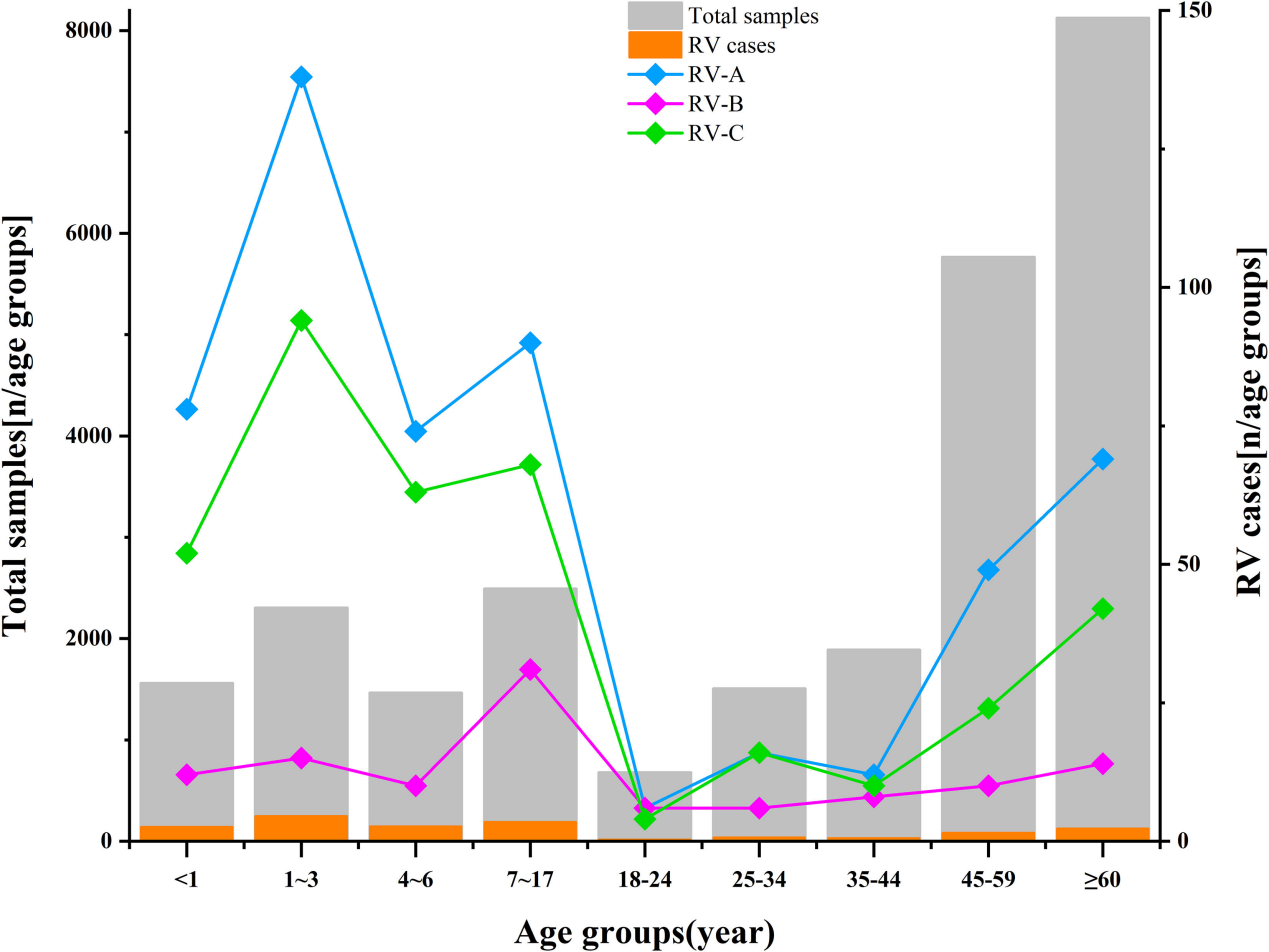
Age distribution of rhinovirus in this study. The length of the gray bars indicates the total number of samples in each age group. The length of the orange bars indicates the number of RV cases detected in each age group. The line chart represents the detection frequency of RV in different age groups (RV-A in blue, RV-B in purple, RV-C in green). Two y-axes are used: the left axis represents the total samples in this study, while the right axis indicates the number of detected rhinoviruses in each age group.

**Figure 6:**
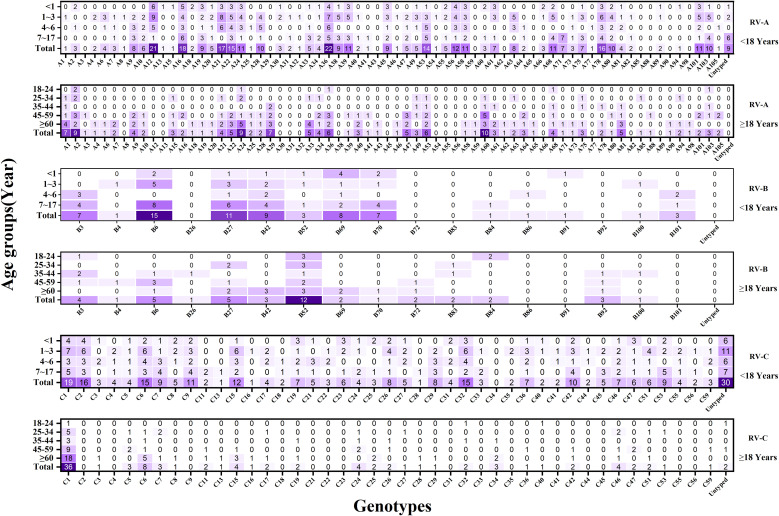
Heat map of the total number of rhinovirus genotypes detected in each age group during the study period (n = 1017). The x-axis represents the detection of different RV genotypes, and the y-axis represents the detection of various RV genotypes in different age groups. Darker colors indicate higher detection frequencies.

Adult RV prevalence was significantly lower than that observed in children, with adults showing a detection rate of 1.63% (292/17,957). The predominant genotypes identified in adults were C1, B52, and A60. By comparison, children demonstrated an overall detection rate of 9.28% (725/7,812), with the highest rates occurring in children aged 1–3 years (10.73%, 247/2,302), followed by those aged 4–6 years (10.05%, 147/1,462). The predominant genotypes in children—C-untyped, A36, and A12—differed entirely from those identified in adults—C1, B52, and A60. See **Figures 5**, **6**, and **Supplementary Table 2** for detailed data.

In this study, 24.98% (254/1017) of RV-positive cases were co-infected with other pathogens. Among them, there were 127 cases of viral co-infection (50%), 121 cases of bacterial co-infection (47.64%), and 6 cases of fungal co-infection (2.36%). Seven patients (2.76%) had co-infection with two types of pathogens (including 5 cases of triple viral co-infection and 2 cases of bacterial and viral triple co-infection). Nine types of co-infecting viruses were detected, with the top five co-infection rates in the following descending order: ADV > RSV > Boca > MPV > PIV; 17 types of co-infecting bacteria were detected, with the top five co-infection rates in the order of: *Mycoplasma pneumoniae* > *Streptococcus pneumoniae* > *Haemophilus influenzae* > *Staphylococcus aureus* > *Klebsiella pneumoniae*; one type of co-infecting fungus was detected: *Candida* spp. No cases of co-infection involving two distinct RV genotypes were identified (see **Table 2**).

**Table 2:** Distribution of co-infecting pathogens.

Viruses	n()	Bacteria	n(%)	Fungi	n(%)
Respiratory syncytial virus	30 (11.81)	*Mycoplasma Pneumoniae*	41 (16.14)	*Candida* spp	6 (2.36)
Adenovirus	30 (11.81)	*Streptococcus pneumoniae*	16 (6.30)		
Bocavirus	20 (7.87)	*Haemophilus influenzae*	14 (5.51)		
Metapneumovirus	19 (7.48)	*Staphylococcus aureus*	12 (4.72)		
Parainfluenza virus	18 (7.08)	*Klebsiella pneumoniae*	9 (3.54)		
Influenza virus type B	5 (1.97)	*Acinetobacter baumanii*	6 (2.36)		
Coronavirus	5 (1.97)	*Escherichia coli*	6 (2.36)		
influenza virus type A -H3N2	3 (1.18)	*Pseudomonas aeruginosa*	4 (1.57)		
influenza virus type A -H1N1	2 (0.79)	*Chlamydia*	4 (1.57)		
		*Burkholderia cepacia*	2 (0.79)		
		*Enterobacter cloacae*	1 (0.39)		
		*Listeria monocytogenes*	1 (0.39)		
		*Moraxella catarrhalis*	1 (0.39)		
		*Stenotrophomonas maltophilia*	1 (0.39)		
		*Streptococcus*	1 (0.39)		
		*Legionella* spp.	1 (0.39)		
		*Morganella Fulton*	1 (0.39)		
		*Serratia marcescens*	1 (0.39)		
		*Acinetobacter*	1 (0.39)		

Pathogens detected by type, with *n* indicating the frequency of detection.

Of the 1,017 RV-positive cases, 25.27% (257/1,017), 42.97% (437/1,017), and 31.76% (323/1,017) were diagnosed with URTI, LRTI, and remained undiagnosed, respectively. Within both URTI and LRTI groups, RV-A emerged as the most frequently detected species, followed by RV-C. The distribution patterns among the three groups revealed statistically significant differences (P < 0.05). RV-A infections demonstrated more frequent associations with fever, cough, wheezing, and dyspnea than did RV-C and RV-B. Conversely, RV-C infections showed stronger associations with rhinorrhea and convulsions, whereas RV-B was predominantly associated with cough. Rash represented the least common symptom overall and occurred primarily in RV-C infections. The genotypes most frequently associated with wheezing, bronchopneumonia, and asthma exacerbations in children comprised A24, A36, A21, A22, A12, C1, C-untyped, C6, A16, A56, C15, and C9. Thirteen patients (1.28%) required intensive care unit (ICU) admission, the majority being adults with RV-A infection who presented with concurrent co-infections. The most frequently identified genotypes in ICU cases were C1 and A40. Laboratory analyses revealed that RV-C infections produced the highest elevations in C-reactive protein (CRP) and procalcitonin (PCT) levels, with statistically significant differences observed among the three RV species (P < 0.05). No significant differences emerged in other laboratory parameters (see **Tables 1**, **3**, **Supplementary Figure**).

**Table 3:** Comparison of samples with single infection and co-infections.

Characteristics		RV only	RV coinfect	Total	*P*
Positive	[% (n/total)]	2.96 (763/25769)	0.99 (254/25769)	3.95 (1017/25769)	**0.000**
Season					
Before COVID-19	[% (n/total)]	2.49 (23/925)	0.22 (2/925)	2.70 (25/925)	**0.000**
During COVID-19	[% (n/total)]	3.67 (443/12065)	1.24 (150/12065)	4.92 (593/12065)	**0.000**
After COVID-19	[% (n/total)]	2.32 (297/12779)	0.78 (102/12779)	3.12 (399/12779)	**0.000**
Study population					
female	[% (n/total)]	2.79 (317/11356)	0.85 (96/11356)	3.64 (413/11356)	**0.000**
male	[% (n/total)]	3.09 (446/14413)	1.10 (158/14413)	5.32 (604/14413)	**0.000**
length of stay (days)	median (IQR)	8 (6-11)	10 (7-12)	8 (6-11)	**0.009**
ICU stay	[median range]	0.39 (4/1017)	0.88 (9/1017)	1.28 (13/1017)	0.164
RV species					
RV-A	[% (n/total)]	1.58 (408/25769)	0.48 (124/25769)	2.06 (532/25769)	**0.000**
RV-B	[% (n/total)]	0.31 (80/25769)	0.12 (32/25769)	0.43 (112/25769)	**0.000**
RV-C	[% (n/total)]	1.07 (275/25769)	0.38 (98/25769)	1.45 (373/25769)	**0.000**
Clinical presentation					
fever	[% (n/total)]	29.89 (304/1017)	12.49 (127/1017)	42.38 (431/1017)	**0.000**
Cough	[% (n/total)]	41.00 (417/1017)	15.83 (161/1017)	56.83 (578/1017)	**0.000**
runny nose	[% (n/total)]	8.16 (83/1017)	3.74 (38/1017)	11.90 (121/1017)	**0.000**
rhinobyon	[% (n/total)]	3.64 (37/1017)	1.38 (14/1017)	5.01 (51/1017)	**0.001**
pharyngalgia	[% (n/total)]	4.03 (41/1017)	0.88 (9/1017)	4.92 (50/1017)	**0.000**
wheeze	[% (n/total)]	6.00 (61/1017)	1.97 (20/1017)	7.96 (81/1017)	**0.000**
dyspnea	[% (n/total)]	3.93 (40/1017)	1.08 (11/1017)	5.01 (51/1017)	**0.000**
arthralgia	[% (n/total)]	1.08 (11/1017)	1.08 (11/1017)	2.16 (22/1017)	1.000
convulsion	[% (n/total)]	1.77 (18/1017)	0.79 (8/1017)	2.56 (26/1017)	**0.048**
rash	[% (n/total)]	0.69 (7/1017)	0.19 (2/1017)	0.88 (9/1017)	0.095
Diagnosis					
URTI	[% (n/total)]	20.06 (204/1017)	5.21 (53/1017)	25.27 (257/1017)	**0.000**
pharyngitis	[% (n/total)]	9.05 (92/1017)	2.06 (21/1017)	11.11 (113/1017)	**0.000**
tonsillitis	[% (n/total)]	7.57 (77/1017)	2.26 (23/1017)	9.83 (100/1017)	**0.000**
rhinitis	[% (n/total)]	2.26 (23/1017)	0.59 (6/1017)	2.85 (29/1017)	**0.001**
laryngitis	[% (n/total)]	1.18 (12/1017)	0.29 (3/1017)	1.47 (15/1017)	**0.020**
LRTI	[% (n/total)]	28.22 (287/1017)	14.75 (150/1017)	42.97 (437/1017)	**0.000**
pneumonia	[% (n/total)]	17.90 (182/1017)	10.42 (106/1017)	28.32 (288/1017)	**0.000**
bronchiolitis	[% (n/total)]	7.67 (78/1017)	2.46 (25/1017)	10.13 (103/1017)	**0.000**
asthma	[% (n/total)]	1.57 (16/1017)	1.18 (12/1017)	2.75 (28/1017)	0.447
COPD	[% (n/total)]	1.08 (11/1017)	0.69 (7/1017)	1.77 (18/1017)	0.344
Undefined	[% (n/total)]	26.75 (272/1017)	5.01 (51/1017)	31.76 (323/1017)	**0.000**

URTI, upper respiratory tract infection; LRTI, lower respiratory tract infection; COPD, chronic obstructive pulmonary disease; HRV, human rhinovirus; CRP, C-reactive protein; PCT, procalcitonin; ICU, Intensive Care Unit. IQR: inter-quartile range.

*Statistical significance: p < 0.05. The bold values represent significant results with P < 0.05.

## 4 Discussion

The global COVID-19 pandemic has underscored the critical importance of understanding and predicting respiratory virus behavior. Although traditionally viewed as the primary cause of the common cold, RV has received relatively limited research attention. However, accumulating evidence demonstrates that RV represents not only a major cause of influenza-like upper respiratory tract illnesses but also a significant pathogen in LRTIs, particularly among infants, older individuals, and immunocompromised patients, who face elevated risks for severe disease (Custovic et al., 2018; Bergroth et al., 2020). Kunming, situated in the southwestern border of China, experiences a temperate climate throughout the year and functions as a strategic gateway linking Southeast Asian countries with inland China. To examine the genetic diversity of RV in this region, we undertook a comprehensive study from 2019 to 2023 in collaboration with a major general hospital in Kunming.

This study successfully identified 1,017 RV-positive cases among 25,769 samples, yielding an overall detection rate of 3.95%. The highest detection rate was observed in children, consistent with findings from France, Shanghai, and Slovenia (Kenmoe et al., 2021; Jiang et al., 2022; Berginc et al., 2024). These findings suggest that immature immune systems contribute to the widespread circulation of RV among younger children, indicating the need for enhanced routine preventive measures. The study population consisted primarily of hospitalized patients, among whom the RV detection rate (4.29%) was notably higher than that among outpatients (1.78%), suggesting that RV infection contributes to increased hospitalization rates and the overall healthcare burden. Notably, the median age of infection varied by RV genotype, with RV-B infections observed at the highest median age, indicating that RV-B predominantly circulates among older individuals, whereas RV-A and RV-C were more prevalent among younger children.

With increasing global population mobility, RV genotypic diversity has become more pronounced, with RV-A and RV-C emerging as the predominant circulating species, while RV-B remains relatively uncommon (Mubareka et al., 2013). This study similarly found co-circulation of all three RV species, with RV-A and RV-C being the dominant strains. Notably, the predominant circulating strain shifted over the study period, from RV-A during the COVID-19 period to RV-C after COVID-19, suggesting that changes in epidemic prevention and control measures interfere with the prevalence of respiratory pathogens. This enhances our understanding of the interactions between respiratory pathogens. Unexpectedly, 128 distinct RV genotypes were identified, along with 40 untyped strains and one recombinant strain, indicating a level of genotype diversity that surpasses that reported in previously published studies, particularly for RV-A, which accounted for 68 distinct genotypes in this study. Moreover, several common genotypes identified in this study, such as C1, A36, A12, A21, C6, A16, A22, and B6, differed from the globally prevalent strains reported in earlier studies (e.g., A78, C2, and A12) (Zhao et al., 2018; Aizawa et al., 2024). The rich genetic diversity of RV, coupled with the continuous emergence of new strains, indicates that the virus strains are evolving, which poses significant challenges for epidemic prevention and vaccine development.

The absence of proofreading and repair mechanisms in the RNA-dependent RNA polymerase encoded by RV results in an elevated mutation rate, thereby promoting the emergence of novel and diverse genotypes (Kiyota et al., 2013; Tan et al., 2024). Our study identified 40 Untyped strains and one recombinant strain, occurring primarily within RV-C, followed by RV-A, with no such strains detected in RV-B. These variants represented 24.26% (41/169) of all identified genotypes, a proportion consistent with findings from a Shanghai-based study (22.5%) (Bochkov and Gern, 2012). However, the Shanghai study population comprised exclusively patients with severe acute respiratory infections, whereas our study population included individuals presenting with common acute respiratory tract infections. This observation indicates an increasing association between Untyped RV genotypes and severe respiratory disease. The determination of whether the Untyped strains identified in this study constitute region-specific variants or novel genotypes requires further investigation. These mutant and recombinant strains facilitate RV evolution through alterations in virulence, enhanced susceptibility, increased pathogenicity, and elevated mortality risk (Bochkov YA et al., 2012). Continuous surveillance remains essential to prevent potential outbreaks.

Previous studies have confirmed that RV circulates year-round with clear seasonal patterns, typically peaking in spring and autumn (Esneau et al., 2022; Berginc et al., 2024). However, no distinct seasonal trends were observed in the present study. Instead, RV-A and RV-C alternated in predominance during and after the COVID-19 pandemic. Notably, the resurgence of severe acute respiratory syndrome coronavirus 2 during this period may have temporarily reduced host susceptibility to RV, contributing to a sharp decline in RV circulation in January 2023, which subsequently rebounded, reaching a new peak in September with the start of the school term. Additionally, our study found that the predominant genotypes differed markedly between children and adults: C1, B52, and A60 were predominant among adults, whereas untyped strains, A36, and A12 were most common among children. These findings indicate that the COVID-19 pandemic disrupted the typical distribution of RV strains, leading to shifts in dominant genotypes and age-specific patterns of infection, highlighting the complexity of RV epidemiology.

Among the 1,017 RV-positive cases in this study, 42.97% were diagnosed with LRTIs. Several genotypes, including A24, A36, A21, A22, A12, C1, C-untyped, C6, A16, A56, C15, and C9, were closely associated with varying severities of pneumonia, bronchopneumonia, asthma, and chronic obstructive pulmonary disease. Notably, C-untyped strains primarily affected young children, with nearly one-third of these cases ultimately diagnosed with wheezing bronchopneumonia, suggesting increased pathogenicity of these emerging variants. Given that current treatment for acute asthma exacerbations in children relies heavily on corticosteroids, an approach that has remained unchanged for years, accurate RV genotyping is critical for predicting clinical severity. This study also demonstrated that, in addition to upper respiratory tract diseases, there is a rising trend of RV-A causing LRTIs and severe diseases, representing a shift from previous findings (Linder et al., 2013). Patients infected with RV-C showed the most pronounced elevations in PCT and CRP levels, consistent with findings from a study in Virginia, USA (Teague et al., 2024), indicating a more pronounced inflammatory response to RV-C infections. No outbreaks or mortality were observed throughout the study period.

RV infection disrupts the respiratory epithelial barrier, thereby enhancing susceptibility to adhesion and invasion by other pathogens, which renders co-infection a relatively common occurrence in clinical practice. Our study identified 254 co-infection cases, yielding a rate of 24.98%, substantially lower than the 46.8% reported in Shanghai (Zhao et al., 2018). MP emerged as the most frequently co-detected pathogen, followed by ADV and RSV. This distribution pattern contrasts with findings from a 10-year Central European study (Madewell et al., 2023), indicating regional variations in circulating pathogen prevalence. The most frequently identified co-infecting bacteria included *Haemophilus influenzae*, *Streptococcus pneumoniae*, and *Staphylococcus aureus*, findings consistent with previous reports (Yan et al., 2023). These co-pathogens contribute to increased susceptibility to recurrent wheezing and asthma while complicating disease management strategies (Palmu et al., 2019), indicating a correlation between the level of bacterial colonization and severity of acute respiratory infections and disease. Therefore, timely identification of co-infections is crucial to diagnose and treat the disease. Simultaneously, a higher rate of co-infection suggests, to some extent, the synergistic effects of viruses and bacteria at both the epidemiological and individual host levels, which are consistent with the conclusions of the study (Nickbakhsh et al., 2019). A better understanding of the biological mechanisms of the interactions between viruses, bacteria, and virus-bacteria, as well as their impact on the dynamics of infection within the host, merits further investigations.

In summary, this molecular epidemiological study of RV in Kunming from 2019 to 2023 provides a comprehensive understanding of the genetic diversity, along with the complex clinical pathogenicity in the region. This study clarified the genotypes associated with severe disease, which can better predict severity and develop treatment strategies. Additionally, the higher co-infection rates and age-specific genotype patterns provide baseline data for public health interventions and emphasize the importance of ongoing monitoring. It provides valuable data for this multiethnic border region, offering important insights for enhancing the prevention and control of respiratory infections and promoting public health in minority communities, along with the scope for future research. We believe that this work has significant theoretical and practical public health implications.

Strengths of this study:

1. This is the first study in the region investigating ARTIs, with the largest sample size and longest time span to date.
2. The study period encompasses three key phases: before, during, and after the COVID-19 pandemic, allowing for a comparative analysis to objectively evaluate changes in RV epidemiology.
3. The study revealed extensive RV genetic diversity and confirmed that, in addition to RV-C, RV-A is also a significant pathogen of LRTIs in children.

Limitations of this study:

1. Our research started in September 2019, coinciding with the onset of the COVID-19 outbreak in China in January 2020, which disrupted sample collection between January and October 2020, resulting in an imbalance in the cohort data.
2. The detection of an additional pathogen was considered a co-infection; however, particularly for bacterial pathogens, colonization cannot be entirely ruled out.
3. This was a retrospective analysis conducted at a single center, which may limit the generalizability of the findings to other healthcare settings or regions.

## Data Availability

The original contributions presented in the study are included in the article/**Supplementary Material**. Further inquiries can be directed to the corresponding authors.

## Supplementary material

Supplementary Table 1 Monthly distribution of samples included between 2019 and 2023.

Supplementary Table 2 Detection frequency of RV in different age groups. Frequencies of RV in age groups were calculated using the number of patients infected with each genotype as the numerator and the total number of patients in that age group as the denominator. *Statistical significance: p < 0.05. The bold values represent significant results with P < 0.05.

Supplementary Figure 1 Heat map of the distribution of clinical presentation detected by each genotype. The x-axis represents the detection of different RV genotypes, and the y-axis represents the frequency of related clinical presentation in different RV genotypes. Darker colors indicate higher detection frequencies.
