# Supplementary material for: Epidemiological shift and clinical characteristics of rhinovirus genotypes in acute respiratory tract infection cases in Kunming, China, from 2019 to 2023
Source: Front Cell Infect Microbiol. 2025 Nov 14;15:1678343. doi: 10.3389/fcimb.2025.1678343 (PMC12660223; doi:10.3389/fcimb.2025.1678343)
Supplement: Supplementary Table 1 — Monthly distribution of samples included between 2019 and 2023. [file DataSheet1.pdf]

Supplementary Table 1. Monthly distribution of samples included between 2019 and 2023

Years		2019					2020												2021											
Months		A	S	O	N	D	J	F	M	A	M	J	J	A	S	O	N	D	J	F	M	A	M	J	J	A	S	O	N	D
Total samples	[n/Month]	0	66	104	84	140	531	0	0	0	0	0	0	0	0	392	466	473	399	275	390	358	393	411	378	294	303	368	522	648
RV-A	[n/Month]	0	2	4	2	3	4	0	0	0	0	0	0	0	0	5	18	18	13	8	7	16	18	29	20	10	11	21	11	9
RV-B	[n/Month]	0	0	1	1	1	0	0	0	0	0	0	0	0	0	1	1	5	2	0	1	3	0	1	2	1	0	10	8	5
RV-C	[n/Month]	0	0	2	1	3	1	0	0	0	0	0	0	0	0	4	23	14	5	2	17	7	7	6	3	3	2	3	12	17
untyped cases	[n/Month]	0	0	0	0	0	0	0	0	0	0	0	0	0	0	0	5	4	0	0	3	1	0	2	0	0	0	0	0	4
Years		2022												2023																
Months		J	F	M	A	M	J	J	A	S	O	N	D	J	F	M	A	M	J	J	A	S	O	N	D					
Total samples	[n/Month]	582	392	476	348	410	524	599	537	349	365	625	797	806	497	1416	1442	1470	1242	1037	799	725	860	1059	1426					
RV-A	[n/Month]	4	0	6	14	15	17	15	9	11	7	16	3	0	1	4	5	26	20	23	14	29	27	11	26					
RV-B	[n/Month]	4	0	4	4	2	2	2	1	2	3	8	1	1	0	2	3	3	0	0	4	7	7	5	5					
RV-C	[n/Month]	4	1	10	7	7	10	8	1	0	1	12	3	2	4	22	23	18	11	2	10	35	24	10	18					
untyped cases	[n/Month]	2	2	1	0	4	0	1	0	1	0	2	0	3	1	0	1	0	2	0	1	0	0	0	4					

Supplementary Table 2. Detection frequency of RV in different age groups

Age groups	%(n/Total)	RV-A	RV-B	RV-C	*P*
<1	9.11[142/1559]	78	12	52	**0.000**
1~3	10.73[247/2303]	138	15	94	**0.000**
4~6	10.05[147/1642]	74	10	63	**0.000**
7~17	7.79[189/2489]	90	31	68	**0.000**
18-24	2.37[16/676]	6	6	4	0.777
25-34	2.52[38/1505]	16	6	16	0.070
35-44	1.59[30/1888]	12	8	10	0.669
45-59	1.44[83/5764]	49	10	24	**0.000**
⩾60	1.54[125/8124]	69	14	42	**0.000**
⩽18	9.28[725/7812]	380	68	277	**0.000**
⩾18	1.63[292/17957]	152	44	96	**0.000**
≤18/⩾18	9.28/1.63				**0.000**
