# Supplementary figures and images for: Epidemiological shift and clinical characteristics of rhinovirus genotypes in acute respiratory tract infection cases in Kunming, China, from 2019 to 2023
Source: Front Cell Infect Microbiol. 2025 Nov 14;15:1678343. doi: 10.3389/fcimb.2025.1678343 (PMC12660223; doi:10.3389/fcimb.2025.1678343)

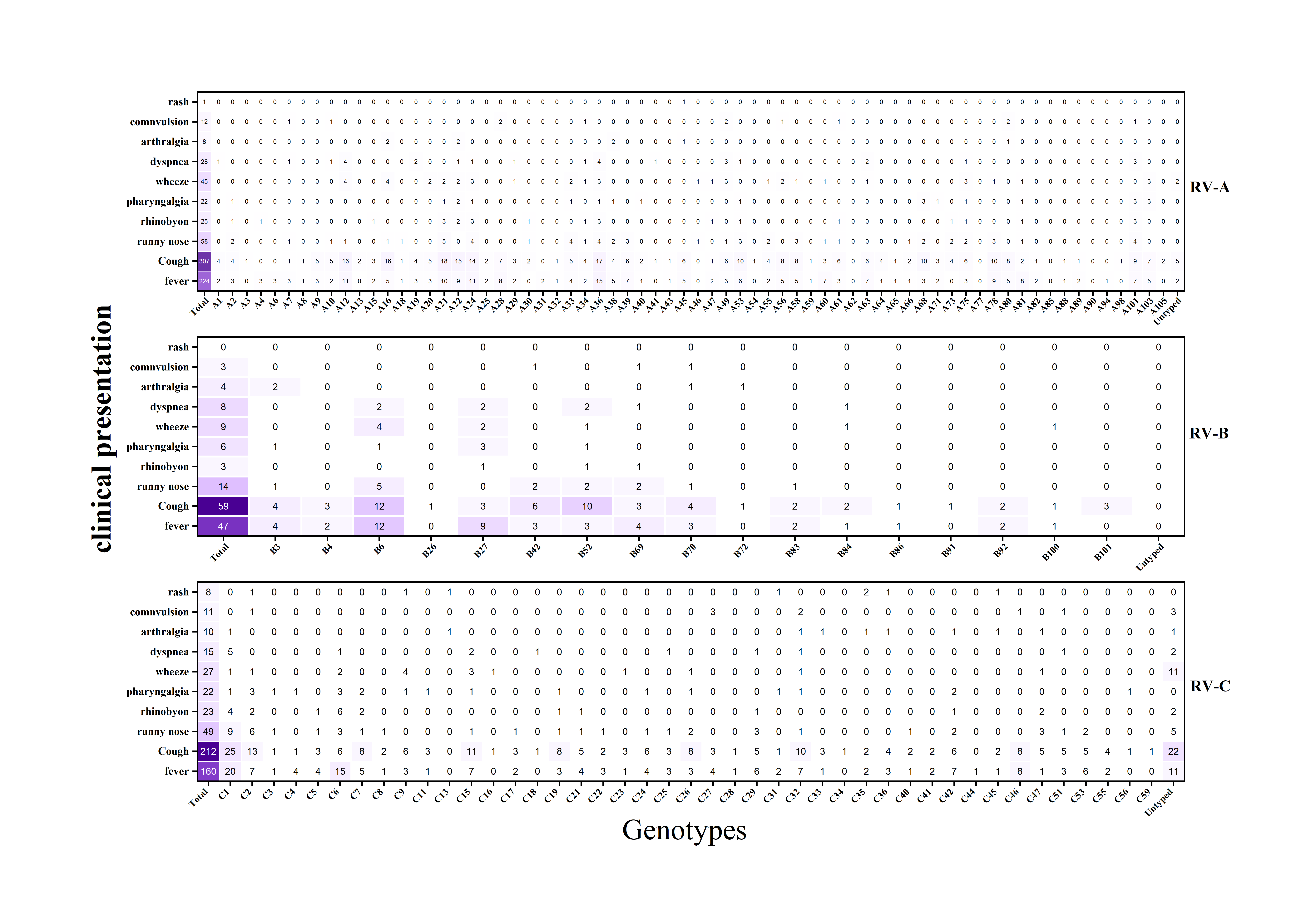

Supplement: Supplementary Figure 1 — Heat map of the distribution of clinical presentation detected by each genotype. The x-axis represents the detection of different RV genotypes, and the y-axis represents the frequency of related clinical presentation in different RV genotypes. Darker colors indicate higher detection frequencies. [file Image1.tif]
